# Study on the Evaluation of Various Types of Sham Acupuncture Treatments in Hemiplegic Stroke Patients: The Protocol of a Randomized Clinical Trial

**DOI:** 10.1155/2019/7395927

**Published:** 2019-10-08

**Authors:** Seungwon Kwon, Seung-Yeon Cho, Seong-Uk Park, Woo-Sang Jung, Sang-Kwan Moon, Jung-Mi Park, Chang-Nam Ko, Ki-Ho Cho

**Affiliations:** Department of Cardiology and Neurology, College of Korean Medicine, Kyung Hee University, Seoul, Republic of Korea

## Abstract

**Background:**

Various sham acupuncture devices have been developed and used in studies on acupuncture. However, there is controversy on whether these devices act as an appropriate placebo or control. In particular, validation of sham acupuncture has only been performed in studies involving healthy individuals. In this regard, questions on the suitability of various sham acupuncture treatments in studies involving disease treatment remain unanswered. Therefore, in this study, we would like to investigate the most appropriate sham acupuncture in the research on treatment of hemiplegic stroke.

**Methods:**

The proposed study is a single-center, prospective, randomized sequence, participant- and assessor-blinded trial with four parallel arms. A total of 90 participants will be randomly assigned to Group 1, Group 2, Group 3, or Group 4 in a 1 : 1 : 1 : 1 ratio. All groups will be treated with Quchi (LI11) for 20 minutes. Group 1 will be treated with verum acupuncture; Group 2, with Park Sham Device; Group 3, with Streitberger's needle; and Group 4, with insertion- and removal-type devices. Participants will undergo one treatment session. The primary outcome is Bang's blinding index. Secondary outcomes are the “Discomfort caused by acupuncture therapy” questionnaire and the Massachusetts General Hospital Acupuncture Sensation Scale index. Immediately after the procedure, all participants will also be monitored for adverse events.

**Discussion:**

This study will help identify the optimal sham acupuncture device that can be used for clinical studies on acupuncture treatment in hemiplegic stroke patients. This trial is registered with KCT0002622.

## 1. Introduction

Acupuncture has been widely used for the treatment of various diseases for decades, not only in Asia but also in the West. Several randomized controlled clinical trials have been conducted to scientifically demonstrate the effectiveness of acupuncture treatments. However, there are conflicting opinions on whether acupuncture treatment has a statistically significant effect in comparison with sham acupuncture (control) in each of the published studies [1]. Several studies have reported that both verum acupuncture and sham acupuncture have a significant therapeutic effect compared with no treatment [2, 3]; in contrast, others have suggested that verum acupuncture is more effective than sham acupuncture [4–6]. There are also some reports that state that verum acupuncture does not have a superior therapeutic effect compared with sham acupuncture [7–9].

Various types of sham acupuncture devices have been used in each study. The most representative type of sham acupuncture device is the Park Sham Device [10], which focuses on the puncture of the needle. In addition, there are other models using Streitberger's needle [11], a needle-foam device [12], and a toothpick [13]. Recently, the insertion and removal model [14] has also been introduced, which distinguishes between verum acupuncture and sham acupuncture, depending on the period of retention of the needle after skin penetration. Finally, the nonacupoint model [15], which focuses on nonacupoints, is also used for sham acupuncture in clinical studies, paying attention to the specificity of the effect on each acupoint.

In the background of the controversy related to the effects of verum acupuncture and sham acupuncture, which can be confirmed through the results of clinical research on various diseases, there is the question “Is the type of sham acupuncture used for each disease suitable for the patient with the disease?” Assessments of the various sham acupuncture treatments have already been conducted [14, 16]. However, these studies have definite limitations because they do not assess sham acupuncture in patients with a specific disease but in healthy individuals without disease. The characteristics of the diseases were not considered in the assessment of sham acupuncture treatment.

In most clinical studies in stroke patients, acupuncture treatment is usually applied to the paralyzed acupoint. However, the paralyzed limbs of stroke patients have complex patterns of muscle tone, muscle strength, and sensory abnormality [17], and there could be differences in the degree of sensitivity during stitching and the discomfort felt during acupuncture treatment compared to those in healthy individuals. Therefore, it is difficult to apply the validation results of sham acupuncture treatments, evaluated in healthy individuals, to stroke patients. However, there has been no study that has evaluated which type of sham acupuncture is suitable for a study population of stroke patients. In addition, even in acupuncture-related clinical trials involving similar stroke types, there are limitations to performing a meta-analysis because of the different types of sham acupuncture treatments used in each study.

In this study, we will evaluate the success rate of blindness and the posttreatment response to sham acupuncture treatments (three types) compared with verum acupuncture treatment in hemiplegic stroke patients. To assess the success blinding rate of various sham acupuncture treatments, a randomized, double-blinded (participants and evaluator), 4 group paralleled clinical trial will be conducted. Through this study, we aim to explore the most appropriate sham acupuncture tool for clinical research on stroke.

## 2. Methods

### 2.1. Objectives

The objectives of this study are to explore the most suitable acupuncture tool for the clinical study of stroke patients. Therefore, the success rate of the blinding will be determined, and the posttreatment response to sham acupuncture treatments (three types) compared with real acupuncture treatments in patients with hemiplegic stroke will be assessed.

### 2.2. Study Setting and Trial Design

We will conduct this study at the Kyung Hee University Korean Medicine Hospital. This study has been approved by the Institutional Review Board of Kyung Hee University Korean Medicine Hospital (KOMCIRB-170717-HR-020). If the protocol requires modifications, it will be approved by the Institutional Review Board of Kyung Hee University Korean Medicine Hospital. Written informed consent will be obtained from all participants by designated clinical research coordinator (CRC). Patient confidentiality will be ensured before, during, and after the trial.

This study will be a single center, prospective, randomized, evaluator-participant double-blinded trial with four parallel arms: arm 1 (Group 1), arm 2 (Group 2), arm 3 (Group 3), and arm 4 (Group 4). Each participants will be randomly assigned.

### 2.3. Inclusion and Exclusion Criteria

Participants who meet the following criteria will be included: (1) 19 years or older; (2) diagnosed with cerebral hemorrhage or cerebral infarction through brain computed tomography (CT) or magnetic resonance imaging (MRI); (3) weakness or hemiparesis or hemiplegia due to cerebral hemorrhage or cerebral infarction, with a Modified Ashworth Scale (MAS) score of 2 points or less; (4) stable vital signs; and (5) voluntary participation in this study.

The following participants are excluded: (1) patients with unconsciousness, aphasia, and cognitive dysfunction (Mini-Mental State Examination-Korean version (MMSE-K) <24); (2) those with a past history of brain disease (e.g., previous stroke, mental illness, consciousness disorder due to head trauma, previous brain surgery, or spastic disease); (3) those with severe heart, liver, or kidney disease or bleeding disorders; (4) those with other serious diseases (e.g., cancer, dementia (Alzheimer's disease or dementia of Lewy body), Parkinson's disease, and Parkinson's syndrome); (5) those with subarachnoid hemorrhage; (6) those who had received acupuncture treatment for hemiplegia, hemiparesis, and weakness owing to previous stroke; (7) those with needle phobia; and (8) pregnant or lactating female patients.

### 2.4. Recruitment Strategies

We plan to recruit participants from among the inpatients through poster advertisement on the bulletin boards of the hospital. Potential participants will call an investigator and be prescreened for eligibility. The participants of this study should be selected from the applicants who meet all the criteria but do not qualify for any of the exclusion criteria. Even if the results of clinical laboratory testing of potential participants show minor transient abnormalities, they could be selected as eligible participants if an investigator determines that such changes have little influence on the study and are not harmful to the participant. Eligible participants will make an appointment. At the first meeting with a CRC, information about the study (risks and benefits of the study) will be provided. After this procedure, potential participants will sign an informed consent form, if they agree to enroll in this study. The registration number of the study is assigned to all eligible participants.

### 2.5. Randomization and Allocation Concealment

After registration, the participants will be randomized to Group 1, Group 2, Group 3, or Group 4. Group 1 will receive verum acupuncture; Group 2 will receive Park Sham Device acupuncture; Group 3 will receive Streitberger's needle acupuncture; and Group 4 will receive insertion- and removal-type acupuncture. In this allocation, the stratified-block randomization will be conducted by an independent CRC. The stratification is performed according to whether there is a sensory disturbance of the affected limb (1: no sensory disturbance; 2: sensory disturbance). Block lengths are eight. Random assignment will be conducted by computer-based randomization. The block random function of R software (version 3.1.2) will be used to generate a random number. The assignment probability of the treatment group and the three control groups is 1 : 1 : 1 : 1. For allocation concealment, an independent CRC places the random assignment code in an impermeable envelope and seals it, and it is then delivered to the practitioner. The practitioner will open the random assignment envelope in front of the participant and assign the participant. After that, practitioners will keep the opened envelopes with themselves.

### 2.6. Blinding

The participants and the evaluator will be blinded in this trial. All participants will be blinded to the types of acupuncture. After the treatment, an independent, blinded evaluator who does not know the group assignment will conduct the outcome evaluation. The researcher who will oversee the statistical analysis will also be blinded so that the treatment for each group remains unknown. However, it is almost impossible to blind the practitioners who conduct acupuncture. Therefore, the practitioner will be forbidden from discussing the type of acupuncture with the participants.

### 2.7. Interventions

#### 2.7.1. Study Flow and Treatment Procedure

Acupuncture treatment will be performed once during the trial. The acupuncture treatment will be carried out by a practitioner with more than 2 years of clinical experience. Before the treatment procedure, the patient's basic characteristics (e.g., age, sex, body mass index (BMI), vital signs, past medical history, drug consumption, education, occupation, alcohol consumption, smoking habit, and stroke type (cerebral infarction or cerebral hemorrhage), MMSE-K, National Institute of Health Stroke Scale (NIHSS) score, and Modified Rankin Scale will be investigated.

**Figure 1:**
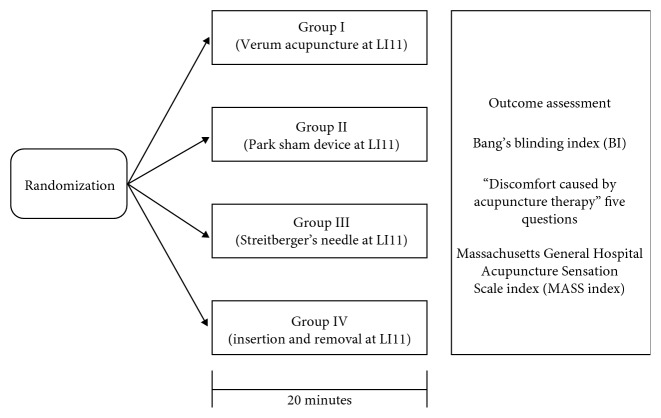
Flow diagram for study. Summary of the study flow.

The entire procedure will be performed over 20 minutes. After all of the preparations for treatment are complete, the participants will be asked to lie on the bed for 2–5 min to stabilize. In the verum acupuncture group, real manual acupuncture will be performed. In the three sham acupuncture groups, Park Sham Device, Streitberger's needle, or insertion- and removal-type acupuncture will be applied. Details of the acupuncture treatment are described in the Standards for Reporting Interventions in Clinical Trials of Acupuncture (STRICTA) checklist in Table 1. During the entire trail period, all the procedures of acupuncture will be carried out by one practitioner who is independent. During the procedure, a prepared visual barrier will prevent participants from viewing their arm and confirming the type of acupuncture. Immediately after the end of the procedure, a questionnaire for determining Bang's blinding index and the “Discomfort caused by acupuncture therapy” questionnaire will be administered, and the Massachusetts General Hospital Acupuncture Sensation Scale (MASS) index, by deqi sensation, will be determined (Figure 1).

#### 2.7.2. Acupuncture Treatment Setting

**Figure 2:**
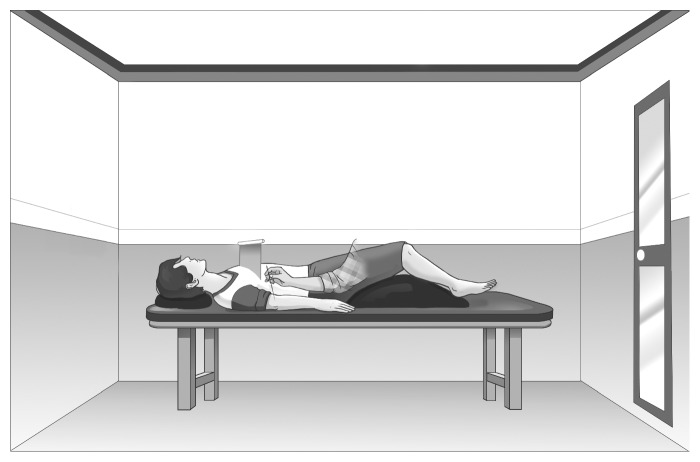
Acupuncture treatment setting.

The acupuncture treatment room setting will be a modification of that in a previous study by Leem et al. [16] and is shown in Figure 2. Participants will lie on the bed in a supine position. A visual barrier will prevent participants from viewing their arm to ensure blindedness.

### 2.8. Outcome Assessments

#### 2.8.1. Primary Outcome

Immediately after the end of the procedure, a questionnaire will be administered to assess the success of blindedness, and the results were used to obtain Bang's blinding index (BI) [18]. The questionnaire is organized as follows: (i) strongly believe the treatment is verum acupuncture, (ii) somewhat believe the treatment is verum acupuncture, (iii) somewhat believe the treatment is sham acupuncture, (iv) strongly believe the treatment is sham acupuncture, and (v) do not know. The BI value ranges from −100 to 100%. A value of 0% represents random guessing (for example, 50% accurate and 50% inaccurate), 100% represents complete unblinding (for example, all answers are accurate), and −100% represents the opposite of guessing (for example, all responses are incorrect).

#### 2.8.2. Secondary Outcome

“*Discomfort Caused by Acupuncture Therapy*” *Five Questions*. On the basis of the results of a previous study [14] on sham acupuncture treatments in healthy individuals, five questions were used to evaluate the discomfort caused by acupuncture therapy. This questionnaire consists of questions on (1) the general perception of acupuncture, (2) discomfort at the moment of puncture, (3) the location of the feeling of puncture, (4) the intensity of discomfort, and (5) the duration of the puncture sensation. Four questions (i.e., general perception of acupuncture, discomfort at the moment of puncture, location of the feeling of puncture, and location of the feeling of puncture) will be answered as either “yes” or “no.” For intensity of discomfort, the 11-point numerical scale, ranging from 0 (no discomfort) to 10 (worst discomfort), will be used. The question on duration of the puncture sensation will be answered in minutes or seconds, and the record will be in seconds.

### 2.9. MASS Index

The MASS index by deqi sensation will also be conducted. It is an index of the 12 total sensations caused by acupuncture (i.e., treatment soreness, aching, deep pressure, heaviness, fullness/distention, tingling, numbness, sharp pain, dull pain, warmth, cold, and throbbing). The MASS index is calculated based on the findings of Kong et al. [19]. Because there is no Korean version, the MASS index, a Japanese version of MASS [20], will be translated and used, as Japan and Korea are countries with very similar cultures and lifestyle.

### 2.10. Sample Size Calculation

This study was not intended to evaluate the effectiveness of acupuncture treatment but rather to evaluate the suitability of various sham devices. Therefore, we did not follow the general method for obtaining the sample size. The target number of participants in this study was selected on the basis of the number of participants in the previous study [14], which was a sham acupuncture comparison study conducted in healthy individuals [14]. In the previous study, the participants were divided into verum acupuncture, Park Sham Device, Needle + Foam, Insertion and Removal, Real + Park Sham device, Real + Needle + Foam, and Real + Insertion and Removal groups. Twenty-two participants per group were recruited. Therefore, on the basis of this, we decided to recruit at least 22 participants for each group and to recruit 90 stroke patients considering withdrawal during the trial.

### 2.11. Statistical Analysis

Hypothesis to be tested in this study is that the certain sham device will reveal the most similar sensation to verum acupuncture in the affected limb of stroke patients. Primary outcome of this study is Bang's blinding index in each group. The value of Bang's blinding index of each sham acupuncture device will be calculated by comparison with the verum acupuncture group. This statistical process will be based on Bang's previous study, published in 2004 [18]. In secondary outcome analysis, the values of “Discomfort caused by acupuncture therapy” five questions and MASS index will be compared by ANOVA. In baseline characteristics, categorial variables will be compared by a chi-square test and continuous variables will be compared by ANOVA. A comparative analysis of the frequency of patients with sensory disturbances by each group will evaluate whether stratification assignments have been made properly. In subgroup analysis, we will compare the values of primary and secondary outcomes in patients with sensory disturbances. Patients with sensory disturbances are defined as patients who complaint sensory disturbances such as numbness, hypesthesia, or paraesthesia in affected limbs. In this study, sensitivity analysis will not be performed. A 5% significance level will be used. Study data will be analyzed using the Statistical Package for the Social Sciences (SPSS) software, version 16.0, for Windows and Microsoft Excel 2010 for windows by a blinded statistician.

### 2.12. Quality Control

For quality control, we will conduct a number of simulations for our colleagues and volunteers before the start of the study. All practitioners will conduct the study according to the standard operating procedure (SOP) of the study. During the course of the study, regular meetings will be held to discuss issues raised by investigators or participants, the need to improve protocols, the side effects, and participant recruitment.

### 2.13. Data Management

Information obtained from each participant will be recorded on a paper document case report form (CRF). The collected information will then be entered into an SPSS file for future statistical analyses with coding. All of the above information will be stored in the confidential computer of the Stroke Center of Kyung Hee University Korean Medicine Hospital. This confidential computer will be managed by the Stroke Center of Kyung Hee University Korean Medicine Hospital. To protect personal information, all participants' full name and resident registration numbers etc. will not be disclosed and a unique identifier number given during the trial will be used to identify participants. All the participants will be informed that the clinical data in this trial will be stored in a confidential computer and will be discarded after the end of the trial. The written consents will be stored by the principal investigator's office in the Stroke Center of Kyung Hee University Korean Medicine Hospital.

### 2.14. Monitoring

A designated CRC among the internal researchers will act as the monitor agent and will compare the data written on the CRFs with those on the source documents. Protocol required data, which are required to fulfill the requirement of the International Conference on Harmonization's Good Clinical Practices (ICH-GCP), will be reported accurately and consistent with the source. In addition, the following aspects will be monitored: modifications of study design, adverse events, and withdrawals. This monitoring will be carried out by the institution itself every six months.

### 2.15. Investigation of Adverse Events and Safety

The type and frequency of adverse reactions in each group will be investigated immediately after a treatment procedure. Any adverse events that occur will be documented, and appropriate treatments for the associated symptoms will be provided.

## 3. Discussion

There is a limitation in this study. All subjects receive only one acupuncture treatment. Most acupuncture clinical trials involve several acupuncture treatments. Therefore, the present study cannot provide insights into the maintenance of blinding over the course of treatment time. Second, the design of this study cannot compare the effects on stroke of three sham acupuncture devices. This design can only evaluate the sensation after puncture of subjects. The effect on the disease is also important for sham acupuncture devices. However, we will not be able to evaluate this point in this study. Third, this study will be conducted as a single center trial.

However, this study would be meaningful in the following points. This study will provide evaluation results on the optimal sham acupuncture device that can be used for clinical studies on acupuncture treatment in hemiplegic stroke patients. The results of this study will be useful for standardizing clinical studies on hemiplegic stroke-related acupuncture in the future. If there are more clinical studies using the same and optimal sham acupuncture device in the future, it will be possible to obtain more definite results than those from the present systematic review and meta-analysis, which comprehensively evaluate the clinical effects of acupuncture in patients with hemiplegic stroke. The results of this study will be published regardless of the outcome, in accordance with the STRICTA guidelines.

## Figures and Tables

**Table 1:** STRICTA checklist (details of intervention).

	Item	Details
1. Acupuncture rationale	Style of acupuncture	Manual acupuncture based on traditional Korean medicine theory
	Reasoning for treatment provided, based on historical context, literature sources, and/or consensus methods, with references wherever appropriate	Acupuncture points to be used in this study were Quchi (LI11) located at the elbow of the affected upper limb. According to a review of systematic literature review and meta-analysis of existing strokes [18], one of the most commonly used acupuncture points for stroke treatment is Quchi
	Extent to which treatment was varied	All participants will receive standardized treatment

2. Details of needling	Number of needle insertions per subject per session	1
	Names of points used	Quchi (LI11) at the affected side
	Depth of insertion, based on a specified unit of measurement	5–10 mm
	Response sought	Simple puncture
	Needle stimulation	Manual acupuncture
	Needle retention time	20 minutes
	Needle type	Group 1: verum acupuncture needle; 0.25 × 40 mm sterilized stainless steel needle (Dong Bang acupuncture, Gyeonggi-do, Korea)

3. Treatment regimen	Number of treatment sessions	1 time
	Frequency and duration of treatment sessions	20 minutes for 1 time

4. Other components of treatment	Details of other interventions administered to the acupuncture group	None
	Setting and context of treatment, including instructions to practitioners, and information and explanations to patients	The study will be conducted in the Kyung Hee University Korean Medicine Hospital and all information will be provided to the subjects

5. Practitioner background	Description of participating acupuncturists	A KMD after completing 6 years of Korean medicine undergraduate course or KM internal medicine specialists who have at least two years of clinical experience

6. Control or comparator interventions	Rationale for the control or comparator in the context of the research question, with sources that justify this choice	Validated placebo acupuncture devices will be used in control groups
	Precise description of the control or comparator. If sham acupuncture or any other type of acupuncture-like control is used, provide details as for items 1 to 3 above	The allocation for control groups (Groups 2∼4) will be decided by randomization (Group 2) 1) Park Sham Device: 0.25 × 40 mm sterilized stainless steel needle (Park Sham Device, AcuPrime, Exeter, UK) will be used Group 3: Real Streitberger's needle; 0.25 × 40 mm sterilized stainless steel needle (Asia-med GmbH & Co. KG, Kirchplatz 1, Germany) Group 4: verum acupuncture needle; 0.25 × 40 mm sterilized stainless steel needle (Dong Bang Acupuncture, Gyeonggi-do, Korea). After puncture, remove immediately without retention The appearance, acupuncture point, stimulation method, and duration will be the same as the real acupuncture group (Group 1) Besides using sham needle tools, all procedures are the same, including acupuncture therapy, stimulation method, and frequency of stimulation

STRICTA: Standards for Reporting Interventions in Clinical Trials of Acupuncture.

## Data Availability

No data were used to support this study.

## Abbreviations

ANOVA: Analysis of variance
BI: Bang's blinding index
BMI: Body mass index
CRC: Clinical research coordinator
CT: Computed tomography
MAS: Modified Ashworth Scale
MASS: Massachusetts General Hospital Acupuncture Sensation Scale
MMSE-K: Mini-Mental State Examination-Korean version
MRI: Magnetic resonance imaging
NIHSS: National Institute of Health Stroke Scale
SOP: Standard operating procedure
SPSS: Statistical package for the social sciences
STRICTA: Standards for Reporting Interventions in Clinical Trials of Acupuncture.

## Additional Points

Recruitment began in October 2017.
